# Supplementary material for: A DNA-Modified Live Vaccine Prime–Boost Strategy Broadens the T-Cell Response and Enhances the Antibody Response against the Porcine Reproductive and Respiratory Syndrome Virus
Source: Viruses. 2019 Jun 14;11(6):551. doi: 10.3390/v11060551 (PMC6630347; doi:10.3390/v11060551)
Supplement: Supplementary file 1 [file viruses-11-00551-s001.pdf]

viruses

MDPI

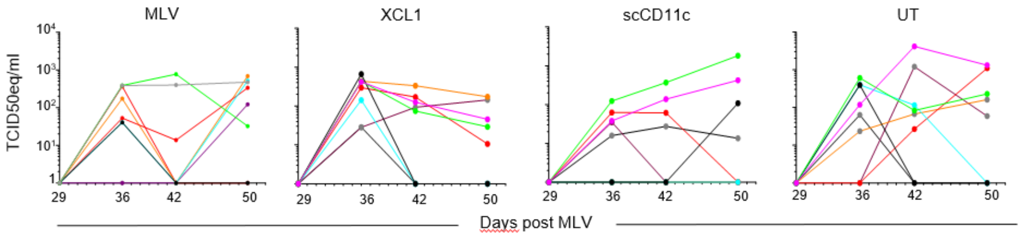

Supplementary Figure 1. Detection of MLV FL13b RNA in sera. The sera of the 4 DNA+MLV groups, collected on D29, 36, 42 and 50, were assayed for detection of MLV FL13b RNA using TaqMan qRT-PCR. TCID50eq/ml were calculated. The extrapolated TCID50eq/ml curves are shown for each pig represented by a distinct color.
